# The Smell of Age: Perception and Discrimination of Body Odors of Different Ages

**DOI:** 10.1371/journal.pone.0038110

**Published:** 2012-05-30

**Authors:** Susanna Mitro, Amy R. Gordon, Mats J. Olsson, Johan N. Lundström

**Affiliations:** 1 Monell Chemical Senses Center, Philadelphia, Pennsylvania, United States of America; 2 Swarthmore College, Swarthmore, Pennsylvania, United States of America; 3 Section of Psychology, Dept. Clinical Neuroscience, Karolinska Institute, Stockholm, Sweden; 4 Department of Psychology, University of Pennsylvania, Pennsylvania, United States of America; Technical University of Dresden Medical School, Germany

## Abstract

Our natural body odor goes through several stages of age-dependent changes in chemical composition as we grow older. Similar changes have been reported for several animal species and are thought to facilitate age discrimination of an individual based on body odors, alone. We sought to determine whether humans are able to discriminate between body odor of humans of different ages. Body odors were sampled from three distinct age groups: Young (20–30 years old), Middle-age (45–55), and Old-age (75–95) individuals. Perceptual ratings and age discrimination performance were assessed in 41 young participants. There were significant differences in ratings of both intensity and pleasantness, where body odors from the Old-age group were rated as less intense and less unpleasant than body odors originating from Young and Middle-age donors. Participants were able to discriminate between age categories, with body odor from Old-age donors mediating the effect also after removing variance explained by intensity differences. Similarly, participants were able to correctly assign age labels to body odors originating from Old-age donors but not to body odors originating from other age groups. This experiment suggests that, akin to other animals, humans are able to discriminate age based on body odor alone and that this effect is mediated mainly by body odors emitted by individuals of old age.

## Introduction

Body odor’s chemical complexity [1] enables it to convey a plethora of biological and social information. In human and non-human animals alike, signals hidden within the body odor cocktail have been suggested to aid in mate selection [2], [3], [4], [5], individual recognition [6], [7], [8], [9], kin detection [2], [10], [11], [12], [13], [14], [15], and sex-differentiation [16], [17], to name a few [18].

There is mounting evidence that body odors also carry age-related information and that animals are able to accurately detect and process that information. It has long been known that the chemical composition of body odors changes in an age-dependent manner in a variety of non-human animals, such as mouse [19], [20], [21], black-tailed deer [22], rabbit [23], otter [24], and owl monkey [25].

However, some of these studies compared very young and adult animals, leaving the unexplored possibility that the demonstrated findings were mediated by a difference in diet; for example, the young animals may still have been nurtured entirely or partly by breast feeding. Indeed, diet is known to affect the chemistry and perception of body odors [26]. In addition, none of these studies demonstrated an ability to differentiate between body odors of different-aged conspecifics. In contrast, Osada and colleagues [20] demonstrated that mice can discriminate between adult and old-age conspecifics based on body odor alone and that this effect was mediated by differences in the quality, rather than the intensity, of the body odors. Together, this evidence suggests that several non-human animal species have the ability to process the age-dependent signals in body odor, and a few studies have even demonstrated that human participants are able to discriminate between animals of different ages based on their body odors alone [23], [24]. Nevertheless, whether humans, like mice, have the ability to infer the age of conspecifics based on body odors alone remains unanswered.

Reported personal observations indicate that human body odors change throughout the life cycle. It is commonly said that old-age individuals have a characteristic body odor, the so-called “nursing home smell” or “old people smell,” an observation that seems to be culture-independent. In humans, dermal body odors originate from a complex interaction between skin gland (eccrine, sebaceous, apocrine) secretions and bacterial activity [27], and skin gland composition and secretion change in an age-dependent manner throughout development. The sebaceous gland is found over much of the skin’s surface and secretes a complex mixture of lipids (sebum) and fatty acids [28], both important precursors to human dermal body odor [29]. In contrast to the eccrine gland (the so-called ‘sweat gland’), the sebaceous gland is less active in young age, reaches peak activity in adulthood, and sharply returns to low activity in the mid-to-late portion of the seventh decade of life [30]. The apocrine glands demonstrate a similar, age-dependent functionality [28]. As a direct reflection of the sebaceous gland’s activity, the skin’s fatty acid composition and variation demonstrate a large degree of similarity between young and very old individuals [31]. To date, two chemically-related compounds have been confirmed to vary with age in humans: nonenal [32] and nonanal [33]. Both compounds increase with age, particularly older individuals, who exhibit a sharp increase in concentration. Thus, taken together, the age-dependent glandular changes and resulting secretory changes, as well as changes in individual chemical components of the dermal body odor mixture, suggest that the needed chemical precursors for behavioral discrimination between age groups based on body odors exists in humans.

Based on the clear evidence from the non-human animal literature and the demonstrated age-dependent differences in human body odor chemistry, we assessed whether humans are able to extract and process age-dependent signals in body odors sampled from conspecifics. To this end, we collected body odors from donors representing three distinctly separate age categories: Young (20–30 years); Middle-age (45–55 years); and Old-age (75–95 years) adults. Young research participants then attempted to discriminate between age categories in a side-by-side comparison, to group them according to age, as well as rate their perceptual properties. We tested two specific hypotheses using forced-choice discrimination and a labeled group test: individuals (1) can discriminate between body odors based on age of the donors and (2) can correctly assign an age group label to body odors.

## Results

### Perceptual Ratings

**Figure 1:**
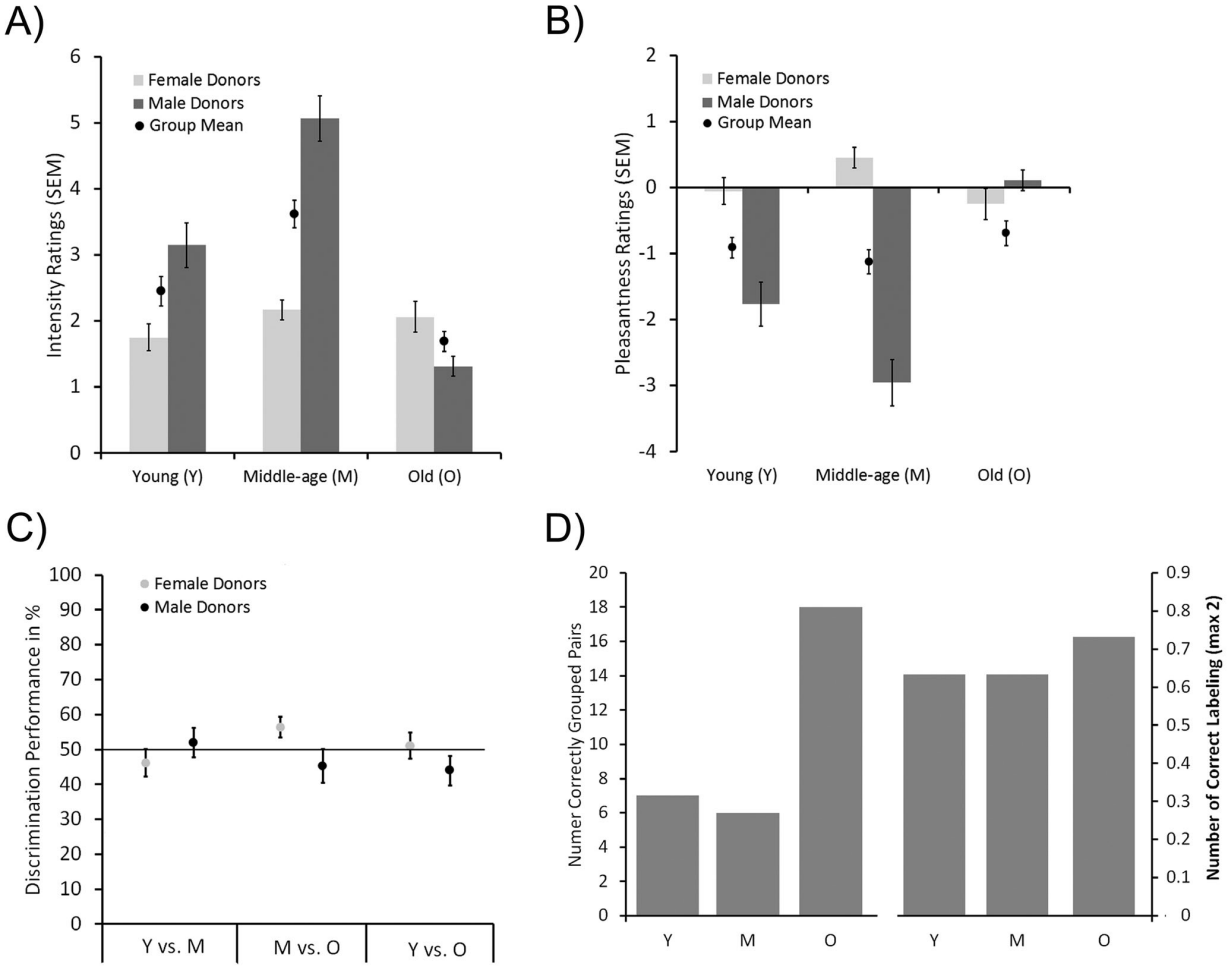
Mean performance for each task. **A**) Mean intensity ratings for each body odor category divided by sex of body odor donor. **B**) Mean pleasantness ratings for each body odor category divided by sex of the body odor donor. Negative values indicate ratings on the unpleasant spectra whereas positive values indicate ratings on the positive spectra. **C**) Mean discrimination performance, measured in percentage correct responses, according to comparison and donor sex. Solid line in graph represents chance performance (50%). **D**) Left hand side indicates total correct pairings of each body odor category and right hand side indicates mean correct age labeling of the body odors. In all graphs, error bars denote standard error of the mean (SEM).

To limit the possibility that potential age-dependent signals would be obfuscated by unknown, individual-specific signals, we created so-called supra-donor stimuli comprised of body odors from multiple individuals of the same age category (see Materials and Methods for a detailed description). Participants initially rated each body odor stimulus’ perceived pleasantness using visual analog scales [34], and intensity using labeled magnitude scale [35], where high values indicate pleasant and intense, respectively. There was a significant difference in perceived intensity between body odors of the three age groups, *F*(2,76) = 31.32, *p*<.01, with subsequent posthoc tests demonstrating that all three possible comparisons demonstrated significant differences (see Table1 for mean values). As seen in Figure 1A, body odors from the Old-age (O) group were rated as significantly less intense than body odors from both the Middle-age (M; *p*<.01) and the Young (Y; *p*<.03) groups. Body odors from the M donors were rated as more intense than body odors originating from the Y donors (*p*<.01). There was no main effect of participant sex on intensity ratings, *F*(1,38) = .47, *p* = .49; however, there was a significant effect of donor sex, *F*(1,38) = 28.64, *p*<.01 as well as a significant interaction between donor sex and donor age group, *F*(2,76) = 45.64, *p*<.01. Subsequent Bonferroni posthoc tests demonstrated that M males were rated as most intense and O males least intense (Y vs. M: *p*<.01; M vs. O: *p*<.01; Y vs. O: *p*<.01). There were no significant interactions between donor age group and donor sex, *F*(2,76) = 2.15, *p* = .12, or between donor sex and participant sex, *F*(1,38) = .26, *p* = .61, with respect to the ratings of perceived body odor intensity.

**Table 1:** Mean values for perceptual ratings and discrimination performance. SEM indicates standard error of the means.

Perceptual Ratings			
Donor Age	Donor Sex	Intensity (SEM)	Pleasantness (SEM)
Y	Female	1.75 (.20)	−0.06 (.20)
	Male	3.14 (.33)	−1.77 (.34)
M	Female	2.17 (.15)	0.45 (.15)
	Male	5.06 (.35)	−2.95 (.35)
O	Female	2.06 (.24)	−0.25 (.24)
	Male	1.31 (.15)	0.10 (.15)
**Discrimination Performance**			
**Group**	**Donor Sex**	**Mean (SEM)**	
Y vs. M	Female	46.11 (3.96)	
	Male	51.94 (4.20)	
M vs. O	Female	56.39 (3.03)	
	Male	45.25 (4.80)	
Y vs. O	Female	51.11 (3.76)	
	Male	43.92 (4.20)	

Although our method of using supra-donor stimuli, in comparison to the method of using stimuli from individual body odor donors, greatly reduces the possibility that body odor of a single donor would mediate the demonstrated effects, one could postulate that the odors of one or two individuals mediated the effects by a potential outlier effect. To ascertain that our effects were not mediated by potential outliers within our supra-donor stimuli, we performed two additional analyses. First, we plotted intensity ratings for each odor category by the participant testing order and smoothed the intensity ratings with a five-subject wide full width at half maximum Gaussian smoothing kernel. The smoothing kernel corresponded to the number of participants for whom an individual odor quadrant was used and was applied to remove individual differences in intensity ratings since these would obscure potential trends in the data. The rationale behind this analysis is that - because odor quadrants were used more or less in the order they were acquired - if one or more odor donors were mediating the demonstrated differences in intensity ratings, there would be a marked difference in ratings when that donor was included in a supra-donor stimulus. As can be seen in Figure S1, there is only one “bump” in the intensity ratings (young males, around testing order positions 29–34). Because supra-donor stimuli were changed after every five subjects, this is the only visually identifiable deviation from the norm. Nevertheless, to investigate this visual effect further, we performed a subsequent outlier analysis of mean intensity ratings using a generalized ESD test for outliers (testing for up to 10 outliers) [36]. This analysis demonstrated that no single supra-donor stimulus could be identified as a statistical outlier (all λs, ns).

The results for the pleasantness ratings were very similar to those from the intensity ratings. There was a significant difference in perceived pleasantness between body odors of the three age categories, *F*(2,76) = 18.16, *p*<.01. Subsequent posthoc tests demonstrated that body odors from O donors were rated as significantly less unpleasant than body odors originating from both M donors (*p*<.01) and Y donors (*p*<.01; see Figure 1B). Body odors from M donors were, however, not perceived as significantly different in pleasantness from Y donors (*p* = .26). As with the intensity ratings, there was no significant main difference in how participating women and men rated the body odors, *F*(1,38) = 1.08, *p* = .31, but there was a significant effect of donor sex, *F*(1,38) = 78.15, *p*<.01, and a significant interaction between donor sex and donor age group, *F*(2,76) = 42.90, *p*<.01. All age groups of male body odor stimuli differed significantly in pleasantness from one another, with M male odor always rated most unpleasant and O male odor most pleasant (Y/M, *p*<.01; M/O, *p*<.01; Y/O, *p*<.01). Among the female body odors, M female odor was rated significantly more pleasant than O female odor (*p* = .01). No other female body odors differed significantly in pleasantness (Y/M, *p* = .08; Y/O, *p* = .75). There were no significant interactions between donor age group and participant sex, *F*(2,76) = .19, *p* = .82, or between donor sex and participant sex, *F*(1,38) = .65, *p* = .42, with respect to the ratings of perceived body odor pleasantness.

### Age Discrimination Task

We assessed ability to discriminate between age groups with a two-alternative, forced-choice test repeated nine times for each age category with the task of determining which one of the two stimuli originated from the older donor. Participants were able to discriminate between body odors of different age categories, as demonstrated by the significant overall main effect of donor age group, *F*(1,64) = 4.54, *p*<.02 (see Table 1 for mean values). There was no main effect of donor sex, *F*(1,32) = .73, *p* = .39, or participant sex, *F*(1,32) = .86, *p* = .36, on participants’ ability to discriminate between the age categories of body odors. However, there was a significant interaction between the factors of donor age group and donor sex, *F*(1.39,32) = 5.29, *p*<.02, indicating that – as with the perceptual ratings – participants’ ability to extract age-dependent information from body odors depends on the sex of the odor donor. There was no significant interaction between donor age and participant sex, *F*(1.71,64) = 1.88, *p* = .16, as well as between donor sex and participant sex, *F*(1,23) = .01, *p* = .91.

Subsequent one-sample Student’s *t*-tests against expected chance performance (50%) demonstrated that participants were significantly able to discriminate M female odors from O female odors (*p*<.04) and Y male odors from O male odors (*p* = .05). However, these significant values did not survive subsequent Bonferroni corrections of the alpha value to adjust for repeated statistical testing. No other comparisons reached significance (Figure 1C).

### Age Labeling Task

When asked to label each body odor according to the three age categories, participants were not able to correctly label either the Y body odors (mean correct.63 out of a total 2) or the M body odors (mean correct.65 out of a total 2), according to χ^2^ contingency tests (both *p*>.10). However, O body odors were correctly labeled (mean correct.74 out of a total 2) significantly more often than expected, according to the χ^2^ contingency tests, χ^2^ (2, 36) = 14.10, *p*<.01; Figure 1D). No sex-dependent differences were observed for this task.

### Implicit Age Categorization Task

Neither Y (mean correct 7) nor M (mean correct 6) body odor stimuli were correctly grouped together more frequently than chance (Y: *p* = .92; M: *p* = .96; Figure 1D). However, O stimuli (mean correct 18) were correctly grouped together significantly more frequently than chance (*p*<.01).

## Discussion

This experiment suggests that, akin to other animals, humans are able to discriminate age based on body odor, alone, and that this effect is mediated mainly by body odors emitted by individuals of old age. The mechanism behind this effect is not currently known, even in non-human animals [20]. Few studies have explored age-related changes in body odor composition, and those few studies have included only a restricted number of participants of the advanced age studied in the experiment [32], [33]. Nevertheless, these studies suggest that elevated levels of certain chemicals are a potential biomarker for old age.

The results of this study support the cross-culturally popular concept of an “old person odor.” Participants were able to discriminate between age groups as well as group the Old-age body odors together significantly more often than expected by chance. Interestingly, the demonstrated ability to discriminate among age groups was mediated entirely by discrimination of the body odors originating from Old-age donors. The age discrimination ability was not, however, a straightforward effect; instead, the interaction between donor age group and donor sex indicated a complicated relationship. It has been demonstrated that many animal species are very good at determining the sex of a conspecific based on body odor alone [37]. Whether humans also have this ability is still under debate. Although several studies have demonstrated that humans indeed have the capacity to accurately determine sex based on body odors sampled from axillary regions [38], [39], [40], palm odors [41], and oral odors [42], assignment of sex seems to be dependent on the perceived intensity and pleasantness of those odors, specifically, intense and unpleasant odors tend to be assigned to the male category [38], [42]. In the present study, there were significant differences in perceptual ratings of body odors originating from male and female donors for all age categories except the Old-age group. These perceptual differences clearly demonstrate that body odors have age-dependent odor characteristics. In addition, participants were able to discriminate between age categories at a higher-than-chance frequency even after variances explained by intensity differences were removed.

The lack of a significant difference in perceived intensity and perceived pleasantness between the body odors of Old-age men and women parallels previous studies. The concentration of lipids present on the skin surface begins to decline to pre-pubescent levels with older age, returning to childhood levels around age 80 [31], suggesting that older men and women share skin chemistry features important for body odor production that are not uniform between the sexes at younger stages of maturity. Moreover, Gallagher and colleagues [33] recently demonstrated that there are no clear differences in whole-body odor composition between elderly men and women. Our finding that body odors originating from Old-age men and women were grouped together in the Implicit Age Categorization task supports these data and suggests that any potential perceptual differences were subservient to the potential age-dependent information.

The body odors donated by the older participants were rated as having a neutral valence. In light of the reports in the popular press where the so-called old age odors are commonly described as unpleasant, this outcome was not predicted. What mediates this discrepancy is not known. However, in everyday life, the old age odor is experienced in the context of an old individual being present. Odor valence ratings are highly dependent in which on the context they are experienced. A recent study demonstrated that the label assigned to an odor is a very important predictor of the rated pleasantness in that a label can turn an unlabeled neutral odor into an odor perceived as very negative [43]. Thus, it is likely that the body odors originating from the old individuals would have been rated as more negative if participants were aware of their true origin.

The ecological relevance of body odor-dependent age discrimination can only be speculated about at this stage. In the non-human animal literature, the ‘good genes’ model [44] has been put forth as an explanation for why female animals are attracted to the odors of older males [20] or why female insects prefer the sex pheromone from older male insects [45]. Signals indicating old age, supposedly regulated by the immune system [2], are favored due to the likelihood that individuals who reach old age possess a strong and adaptive immune system, as well as other adaptive advantages that have allowed them to grow older than their peers. Indeed, older male insects have a higher reproductive success than their younger competitors [44], [45]. According to the standard evolutionary model, reproductive success is a highly sought-after trait. If indeed the age-dependent signals are regulated by the immune system, attempts to dishonestly and prematurely display ‘old age odor signals’ to enhance reproductive success would be associated with a reduction in immune function; this is an elegant means to ensure signal honesty. However, regardless of the biological mechanism regulating these signals, their potential impact in modern human society is likely very limited given the high social value given to visual attributes of age. Although participants were statistically able to discriminate between body odors, as well as able to group them correctly in an age-dependent manner, we want to point out that the nominal effects are modest and participants expressed a low degree of confidence in their abilities.

Studies attempting to assess behavioral relevance of chemical signals in humans using off-site sampling of stimuli, such as the present one, are conceivably affected by numerous factors outside the control of the experimenter. The balance between ecological relevance and stimuli purity is a balance between diametrical aims. Ecological relevance would be maximized when no environmental and behavioral restrictions are enforced upon the body odor donors but relevant signals might be masked by environmental and hygiene odors. Stimulus purity would be maximized if collection took place over weeks in a laboratory environment but donor recruitment and retention would be cumbersome. Although considerable efforts were made to control the collection of body odors within a donor’s home (including detailed t-shirt handling instructions, dietary restrictions, personal hygiene regulations, etc.), differences in lifestyle, living environment, and other factors outside our control might still contribute to the demonstrated age discrimination. We believe, however, that the impact of these variables is minor and counteracted by the use of supra-donors, which minimize any non-age-dependent factors not presented in a majority of the donors. Similarly, due to the scarcity of Old-age body odor donors who were not using regulated pharmaceutical compounds, some individuals in the Old-age donor group did use regular medication. Although none of the medications are known to affect body odor composition, and although there was no difference in perceptual ratings of body odors from individuals using medication and those not using medication, it is still conceivable that this age-dependent discrimination, driven by the Old-age odors, is to some extent mediated by the metabolites of pharmaceutical compounds secreted into the body odors sampled from those elderly donors.

Being the very first study to assess the ability of human participants to determine age from body odors, we focused on a very narrow research question and much remains to be explored. Only young experimental participants were included in this study. It is very much conceivable that the effects demonstrated in this study displays a double age-dependent effect, i.e. that age of the rater has an impact on the ability to determine the age of the body odor donor. Moreover, great care was taken to avoid contamination of exogenous odors, thus lowering the ecological validity of the study. Of interest would be to explore what impact natural masking with hygiene product would have on the demonstrated results.

We can at this point only speculate as to what the potential biological mechanisms could be. It is has been speculated that polymorphonuclear leukocytes [46], a type of white blood cells that demonstrate an age-dependent increase in humans, might be a potential biomarker worth exploring in future studies. Previous studies exploring potential biomarkers of age in human and animal body odors have not been conclusive and often fail to take very old age individuals into account. Nevertheless, identifying potential biomarkers is of great interest and would assist in isolating the underlying biological mechanisms mediating and developing these effects.

In conclusion, these data suggest that, akin to other animals, humans are able to discriminate old individuals from younger individuals based on body odor. The modest effects suggest a limited impact on our everyday interactions but does support previous reports of a unique ‘old person odor’. Further experimental work is clearly warranted to determine the mechanism and function of body odor-dependent age discrimination.

## Materials and Methods

### Ethics Statement

All participants provided written, informed consent prior to participation, and all aspects of the study were approved by the University of Pennsylvania’s Institutional Review Board (IRB) prior to starting the study and performed in accord with the Declaration of Helsinki on Biomedical Studies Involving Human Subjects.

### Participants

A total of 41 healthy participants [21 women; mean age 25.0 years (SD 2.7 years); age range 20–30 years] were included in the final analyses, after the exclusion of four individuals (3 women) characterized as hyposmic based on the clinical norms of the olfactory identification test [47]. No participant donated body odor to the study. The following criteria excluded participation: activly smoking, taking psychopharmacological substances, taking systemic medication (including any hormonal contraceptives), having experienced a head trauma leading to unconsciousness, or self-identifying as anything other than strictly heterosexual. The last restriction was implemented due to previously-demonstrated sexual preference-dependent ratings of body odors [48]. All participants provided written, informed consent prior to participation, and all aspects of the study were approved by the University of Pennsylvania’s IRB.

All included women but five were tested in the follicular phase of their menstrual cycle (day 8–15). Of these five women, three were tested in their menstrual phase (day 1–7) and two in their luteal phase. Dates were defined by post menses onset based on self-report [49].

### Body Odor Donors and Odor Collection

Body odors were sampled from individuals in one of three age groups, ‘Young’ (Y, 20–30y), ‘Middle-age’ (M, 45–55y), or ‘Old-age’ (O, 75–95y). A total of 41 healthy donors, adhering to the same exclusion criteria as the experimental participants, were used. Sixteen individuals (8 women) donated body odor to the Young and Middle-age groups, and 12 individuals (6 women) donated to the ‘Old-age’ group. Donors in the ‘Old-age’ group were permitted to use medication for ailments such as hypertension, cholesterol, and acid reflux, because we were unable to locate a sufficient number of Old-age donors who were not using any compounds classified as FDA-regulated drugs. There are, however, no known reports that these medications alter body odor perception or composition. Donors were selected such that the entire age range of each age group was evenly covered. The samples provided by one Young man, one Middle-age man, and one Old-age man were excluded for smelling of soap or for having a body odor undetectable to the experimenter, which brings those individuals' compliance with the sampling procedures into question. All donors provided written, informed consent prior to participating in the study.

Body odors were collected from donors’ armpits using nursing pads (Ultra-Thin Nursing Pads, Gerber Inc., ON, Canada) sewn into the armpits of t-shirts that had been washed with an odorless detergent before use. This technique has been used successfully in prior studies [4], [14], [50]; the t-shirt serves to both hold the pads in place and protect them from outside contamination. Donors washed their bed linens and towels prior to the odor collection period with the same odorless detergent used for the t-shirts and then wore the t-shirt while sleeping alone at home for five consecutive nights. Before going to bed each night, donors washed their hair and bodies using odorless shampoo and soap to remove residues of exogenous odorous compounds. During the day, donors stored the t-shirts in sealed, odorless plastic bags to protect them from outside contamination. Donors were instructed to refrain from drinking alcohol, smoking, and eating spicy foods and other food products known to be excreted into our body odor for the duration of the odor collection period to avoid altering their natural body odor.

The t-shirt was returned to the experimenter after the fifth consecutive night of odor collection. The resulting body odor containing pads were each evaluated by the experimenter and if any trace of a potential exogenous odor was detected, or the pads were perceived to be lacking a discernible body odor, two additional individuals examined the body odor pads. Pads were included in the study only if the body odor was strong enough to be clearly detected and did not contain any perceivable exogenous odors (such as soap, smoke, perfume/cologne, or alcohol). When not in use, all stimuli were stored in a −80°C freezer to prevent decomposition [51], and stimuli were always handled with disposable, odorless surgical gloves to prevent any possible contamination. Experimental stimuli were subsequently created by cutting each pad into equal size quadrants. These quadrants were used by combining one quadrant from each of four separate same-sex, same-age group individuals into “supra-donor” stimuli [48]. Six supra-donor stimuli (Y, M, and O of each sex) were assembled for each testing session. We used these so-called supra-donor stimuli to remove potential effects mediated by individual odor donors.

### Procedure

To avoid including individuals with olfactory dysfunction, participants’ ability to identify odors was assessed using the Sniffin’ Sticks 16-items Odor Identification test. A score of 10 or lower disqualified individuals with potential hyposmia [47]. After the olfactory identification screening test, participants performed three tasks, a perceptual ratings task, a forced-choice discrimination task, and an age labeling task. Within each task, the six supra-donor stimuli were presented in randomized order using 6 oz., wide-mouth glass jars; the same six odor stimuli were used for the three tasks of a testing session. Pad quadrants were arranged along the walls of the jar so that each pad quadrant was equally exposed. After a total of five subjects had been tested using the same set of stimuli, a new set of stimuli was made to prevent significant deterioration of the signal (see Figure S1). All testing occurred in a room specially designed for human chemosensory testing, which includes a ventilation system that continuously circulates room air to prevent the accumulation of volatiles. In all tests, a minimum inter-trial interval of 30 seconds was enforced between each trial, and breaks were given between each task to minimize odor habituation.

To assess potential differences in perceived pleasantness and intensity between the odor categories, participants rated perceived pleasantness of each body odor stimulus using visual analog scales [34] with the end anchors “Extremely unpleasant” (−5) and “Extremely pleasant” (5). Similarly, participants rated perceived intensity of each body odor stimulus using a labeled magnitude scale [35] with the end anchors “No sensation” (0) and “Strongest imaginable” (10). Stimuli were presented one at a time, and the order of age group presentation was randomized, both within each testing session and between participants.

Ability to discriminate between age groups was assessed with a two-alternative, forced-choice test. Participants were presented with two stimuli originating from different age groups and were asked to determine which of the two body odors originated from the older donor. Body odors were presented one at a time, and the order of age group presentation was randomized, both within each testing session and between participants. Both stimuli of a trial were presented for three seconds, and the second stimulus was presented immediately after the first (approximately 3 s in-between) to minimize the time interval for which odor stimuli needed to be remembered. Participants were not permitted to resample any stimuli. Body odor discrimination was assessed within each sex (Y/M, M/O, Y/O), and these six comparisons were repeated nine times each [52], [53].

The ability to estimate the ages of the body odor donors was also assessed. Participants were presented simultaneously with all six body odor stimuli and were asked to group them according to printed labels placed on the testing room table (“Young”, “Middle-age”, and “Old-age”). No restrictions on time or sampling frequency were given for this Age Labeling task.

Body odors have a very large inter-individual variance, and odor qualities in general, are difficult to assess objectively; indeed, some would say this is impossible. However, the free-sorting nature of the Age Labeling task allowed it to serve a second function: in addition to measuring participants’ ability to consciously label body odors by age, it also allowed us to assess whether the two body odor stimuli belonging to the same age category were grouped together more frequently than expected by chance, independent of whether they were assigned the correct age label or not. If an age group has a characteristic body odor quality, we would expect the two stimuli of that category to be grouped together rather than being assigned separate labels, and we expect that this would be independent of donor sex.

### Statistical Analysis

The results of the discrimination tests were first converted to percentage correct values to allow the use of inference statistics on the underlying binary scale. To assess whether discrimination performance within each odor category differed from chance (50%), we used individual one-sample Student’s *t*-tests of percentage correct discrimination values. Differences in perceptual ratings were analyzed using mixed, repeated-measurements ANOVAs, separately for intensity and pleasantness, using a 2 (participant sex) ×3 (donor age group) ×2 (donor sex) design. Participant sex was entered as a between-subject factor, and donor age group and donor sex were used as within-subject factors. Subsequent Bonferroni posthoc tests were performed to statistically assess differences beyond main effects to control for multiple statistical comparisons. Discrimination performance was converted to a percentage correct discriminations value to correct for dichotomization. To assess statistical differences between stimuli, we used repeated-measurements ANCOVA, organized structurally as described above for perceptual ratings, but with two main differences: First, because our initial analyses of the perceptual ratings demonstrated that the largest perceptual difference between body odor groups was perceived intensity and not perceived pleasantness, intensity ratings for all age and sex groups were entered into the model as covariates (a total of six) to remove variance that could be explained by the intensity ratings. Please note that we refrained from adding the 6 pleasantness ratings as covariance factors because the unduly conservative nature of such analyses (a total reduction of our statistical power with 30% [−12 df]) would create a large risk of producing false negative results and conclusions. Second, because participants’ performance scores demonstrated near-significance on Mauchly’s Test of Sphericity (*p* = .058), all statistical values were submitted to Greenhouse-Geiser correction to avoid false positive results due to a skewed distribution. Age Labeling and Implicit Age Categorization task data (the latter described in detail below) were analyzed using chi-square (χ^2^) contingency tests against a random sampling distribution created using Monte Carlo simulations (n = 1000) within the statistical program R.

## Supporting Information

Figure S1 **Intensity ratings by all individuals of all body odor stimuli.** Smoothed Individual intensity ratings plotted over subject testing order. Y in legend indicates young, first M  =  middle-age, O  =  old, F  =  women, and second M  =  male; meaning that YF denotes ratings of a supra-donor stimuli originating from young women. Intensity ratings were performed on labeled magnitude scales with the end anchors “No sensation” (0) and “Strongest imaginable” (10).
(DOCX)

## References

[1] Labows JN, Reilly JT, Leyden JJ, Preti G, Laden K (1999). Axillary Odor Determination, Formation and Control.. Antiperspirants and Deodorants.

[2] Yamazaki K, Beauchamp GK (2007). Genetic basis for MHC-dependent mate choice.. Adv Genet.

[3] Wedekind C, Seebeck T, Bettens F, Paepke AJ (1995). MHC-dependent mate preferences in humans.. Proc R Soc Lond B Biol Sci.

[4] Lundstrom JN, Jones-Gotman M (2009). Romantic love modulates women’s identification of men’s body odors.. Horm Behav.

[5] Penn D, Potts W (1998). MHC-disassortative mating preferences reversed by cross-fostering.. Proc R Soc Lond B Biol Sci.

[6] Lundstrom JN, Boyle JA, Zatorre RJ, Jones-Gotman M (2008). Functional neuronal processing of body odors differs from that of similar common odors.. Cereb Cortex.

[7] Olsson S, Barnard J, Turri L (2006). Olfaction and Identification of Unrelated Individuals: Examination of the Mysteries of Human Odor Recognition.. Journal of Chemical Ecology.

[8] Bonadonna F, Miguel E, Grosbois V, Jouventin P, Bessiere JM (2007). Individual odor recognition in birds: an endogenous olfactory signature on petrels’ feathers?. J Chem Ecol.

[9] Hepper PG, Wells DL (2010). Individually identifiable body odors are produced by the gorilla and discriminated by humans.. Chem Senses.

[10] Beauchamp GK, Yamazaki K (2003). Chemical signalling in mice.. Biochem Soc Trans.

[11] Eggert F, Muller-Ruchholtz W, Ferstl R (1998). Olfactory cues associated with the major histocompatibility complex.. Genetica.

[12] Mateo JM, Johnston RE (2000). Kin recognition and the ‘armpit effect’: evidence of self-referent phenotype matching.. Proc R Soc Lond B Biol Sci.

[13] Yamazaki K, Beauchamp GK, Curran M, Bard J, Boyse EA (2000). Parent-progeny recognition as a function of MHC odortype identity.. Proc Natl Acad Sci U S A.

[14] Lundstrom JN, Boyle JA, Zatorre RJ, Jones-Gotman M (2009). The Neuronal Substrates of Human Olfactory Based Kin Recognition.. Hum Brain Mapp.

[15] Porter RH, Moore JD (1981). Human kin recognition by olfactory cues.. Physiol Behav.

[16] Baum MJ, Erskine MS, Kornberg E, Weaver CE (1990). Prenatal and neonatal testosterone exposure interact to affect differentiation of sexual behavior and partner preference in female ferrets.. Behav Neurosci.

[17] Sergeant MJ, Dickins TE, Davies NO, Griffiths MD (2007). Women’s Hedonic Ratings of Body Odor of Heterosexual and Homosexual Men.. Archives of Sexual Behavior.

[18] Lundstrom JN, Olsson MJ (2010). Functional neuronal processing of human body odors.. Vitam Horm.

[19] Robinson AB, Dirren H, Sheets A (1976). Quantitative aging pattern in mouse urine vapor as measured by gas-liquid chromatography.. Exp Gerontol.

[20] Osada K, Yamazaki K, Curran M, Bard J, Smith BP (2003). The scent of age.. Proc Biol Sci.

[21] Osada K, Tashiro T, Mori K, Izumi H (2008). The identification of attractive volatiles in aged male mouse urine.. Chem Senses.

[22] Muller-Schwarze D (1971). Pheromones in black-tailed deer (Odocoileus heminonus columbianus).. Anim Behav.

[23] Goodrich BS, Mykytowycz R (1972). Individual and sex differences in the chemical composition of pheromone-like substances from the skin glands of the rabbit, Oryctolagus cuniculus.. J Mammal.

[24] Kean EF, Muller CT, Chadwick EA (2011). Otter scent signals age, sex, and reproductive status.. Chem Senses.

[25] Macdonald EA, Fernandez-Duque E, Evans S, Hagey LR (2008). Sex, age, and family differences in the chemical composition of owl monkey (Aotus nancymaae) subcaudal scent secretions.. Am J Primatol.

[26] Havlicek J, Lenochova P (2006). The effect of meat consumption on body odor attractiveness.. Chem Senses.

[27] Wysocki CJ, Preti G (2000). Human body odors and their perception.. Japanese Journal of Taste and Smell.

[28] Schaal B, Porter RH, Slater PJ, Rosenblatt JS, Beer C, Milinski M (1991). “Microsmatic Humans” Revisited: The Generation and Perception of Chemical Signals.. Advances in The Study of Behavior.

[29] Gower DB, Ruparelia BA (1993). Olfaction in humans with special reference to odorous 16-androstenes: their occurrence, perception and possible social, psychological and sexual impact.. J Endocrinol.

[30] Pochi PE, Strauss JS, Downing DT (1979). Age-related changes in sebaceous gland activity.. J Invest Dermatol.

[31] Nazzaro-Porro M, Passi S, Boniforti L, Belsito F (1979). Effects of aging on fatty acids in skin surface lipids.. J Invest Dermatol.

[32] Haze S, Gozu Y, Nakamura S, Kohno Y, Sawano K (2001). 2-Nonenal newly found in human body odor tends to increase with aging.. J Invest Dermatol.

[33] Gallagher M, Wysocki CJ, Leyden JJ, Spielman AI, Sun X (2008). Analyses of volatile organic compounds from human skin.. Br J Dermatol.

[34] Aitken RC (1969). Measurement of feelings using visual analogue scales.. Proc R Soc Med.

[35] Green BG, Dalton P, Cowart B, Shaffer G, Rankin K (1996). Evaluating the ‘Labeled Magnitude Scale’ for measuring sensations of taste and smell.. Chem Senses.

[36] Rosner B (1983). Percentage Points for a Generalized ESD Many-Outlier Procedure.. Technometrics.

[37] Pierman S, Douhard Q, Balthazart J, Baum MJ, Bakker J (2006). Attraction thresholds and sex discrimination of urinary odorants in male and female aromatase knockout (ArKO) mice.. Horm Behav.

[38] Doty RL, Orndorff MM, Leyden J, Kligman A (1978). Communication of gender from human axillary odors: Relationship to perceived intensity and hedonicity.. Behavioral Biology.

[39] Hold B, Schleidt M (1977). The importance of human odour in non-verbal communication.. Z Tierpsychol.

[40] Russell MJ (1976). Human Olfactory Communication.. Nature.

[41] Wallace P (1977). Individual discrimination of humans by odor.. Physiol Behav.

[42] Doty RL, Green PA, Ram C, Yankell SL (1982). Communication of gender from human breath odors: Relationship to perceived intensity and pleasantness.. Hormones and Behavior.

[43] Djordjevic J, Lundstrom JN, Clement F, Boyle JA, Pouliot S (2008). A rose by any other name: would it smell as sweet?. J Neurophysiol.

[44] Brooks R, Kemp DJ (2001). Can older males deliver the good genes?. Trends Ecol Evol.

[45] Nieberding CM, Fischer K, Saastamoinen M, Allen CE, Wallin EA (2012). Cracking the olfactory code of a butterfly: the scent of ageing..

[46] Olsen RL, Little C (1983). Purification and some properties of myeloperoxidase and eosinophil peroxidase from human blood.. Biochem J.

[47] Hummel T, Kobal G, Gudziol H, Mackay-Sim A (2007). Normative data for the “Sniffin’ Sticks” including tests of odor identification, odor discrimination, and olfactory thresholds: an upgrade based on a group of more than 3,000 subjects.. Eur Arch Otorhinolaryngol.

[48] Martins Y, Preti G, Crabtree CR, Runyan T, Vainius AA (2005). Preference for human body odors is influenced by gender and sexual orientation.. Psychol Sci.

[49] Lundstrom JN, McClintock MK, Olsson MJ (2006). Effects of reproductive state on olfactory sensitivity suggests odor specificity.. Biological Psychology.

[50] Lundstrom JN, Boyle JA, Zatorre RJ, Jones-Gotman M (2008). Functional Neuronal Processing of Body Odors Differ From That of Similar Common Odors.. Cereb Cortex.

[51] Lenochova P, Roberts SC, Havlicek J (2008). Methods of Human Body Odor Sampling: The Effect of Freezing..

[52] Lundstrom JN, Goncalves M, Esteves F, Olsson MJ (2003). Psychological effects of subthreshold exposure to the putative human pheromone 4,16-androstadien-3-one.. Horm Behav.

[53] Lundstrom JN, Olsson MJ (2005). Subthreshold amounts of social odorant affect mood, but not behavior, in heterosexual women when tested by a male, but not a female, experimenter.. Biological psychology.

