# Supplementary material for: The Smell of Age: Perception and Discrimination of Body Odors of Different Ages
Source: PLoS One. 2012 May 30;7(5):e38110. doi: 10.1371/journal.pone.0038110 (PMC3364187; doi:10.1371/journal.pone.0038110)

Supporting information to:

**The smell of age: perception and discrimination of body odors of different ages**

Susanna Mitro^1,2^, Amy R. Gordon^1,3^, Mats J. Olsson^3^, and Johan N. Lundström*^1,3,4^

^1^ Monell Chemical Senses Center, Philadelphia, PA, USA

^2^ Swarthmore College, Swarthmore, PA, USA

^3^ Section of Psychology, Dept. Clinical Neuroscience, Karolinska Institute, Stockholm, Sweden

^4^ Department of Psychology, University of Pennsylvania, PA, USA

**Supplementary Figure S1:** Intensity ratings by all individuals of all body odor stimuli. Smoothed Individual intensity ratings plotted over subject testing order. Y in legend indicates young, first M = middle-age, O = old, F = women, and second M = male; meaning that YF denotes ratings of a supra-donor stimuli originating from young women. Intensity ratings were performed on labeled magnitude scales with the end anchors "No sensation" (0) and "Strongest imaginable" (10).


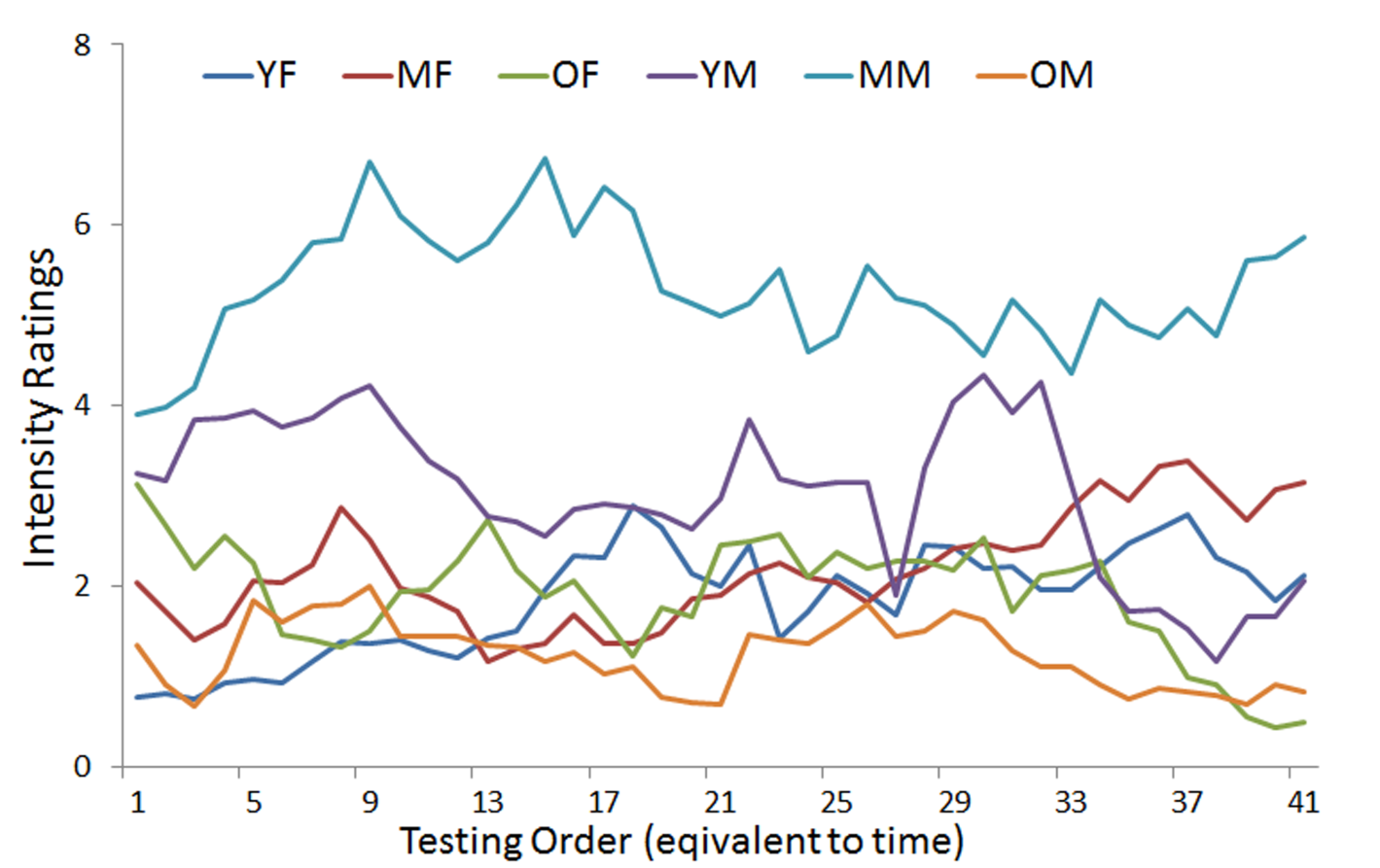

Supplement: Figure S1 — Intensity ratings by all individuals of all body odor stimuli. Smoothed Individual intensity ratings plotted over subject testing order. Y in legend indicates young, first M = middle-age, O = old, F = women, and second M = male; meaning that YF denotes ratings of a supra-donor stimuli originating from young women. Intensity ratings were performed on labeled magnitude scales with the end anchors “No sensation” (0) and “Strongest imaginable” (10). (DOCX) [file pone.0038110.s001.docx]
